# Recovery in schizophrenia: conceptualization and factors implicated

**DOI:** 10.1192/j.eurpsy.2024.63

**Published:** 2024-08-27

**Authors:** A. Vita

**Affiliations:** Department of Experimental and Clinical Sciences, University of Brescia, Brescia, Italy

## Abstract

**Abstract:**

Schizophrenia has a heterogeneous range of possible outcomes. A portion of patients with schizophrenia significantly improves over the long term, with both clinical and functional remission. Recovery has been differently conceptualized by clinicians and service users, the former focusing on clinical and functional outcomes, the latter more underlying issues as the building a trail of personal meaning and subjective well-being. Besides the “clinical” and “personal” recovery, attention is now put on a wider perspective of “societal” recovery . The frequency of recovery achievement depends on which of these perspectives is considered. Many factors, demographic, clinical, contextual and treatment-related are involved in modulating the probability to meet these objectives. Both pharmacological and psychosocial interventions, and their integration, and attention to environmental and social circumstances could substantially improve the outcome of schizophrenia and achievement of specific recovery goals.

**Disclosure of Interest:**

None Declared

